# Are there differences between Mediterranean diet and the consumption of harmful substances on quality of life?—an explanatory model in secondary education regarding gender

**DOI:** 10.3389/fnut.2023.1283195

**Published:** 2023-10-31

**Authors:** Eduardo Melguizo-Ibáñez, Félix Zurita-Ortega, José Luis Ubago-Jiménez, Georgian Badicu, Fatma Hilal Yagin, Gabriel González-Valero, Luca Paolo Ardigò, Pilar Puertas-Molero

**Affiliations:** ^1^Department of Didactics of Musical, Plastic and Corporal Expression, University of Granada, Granada, Spain; ^2^Department of Physical Education and Special Motricity, Faculty of Physical Education and Mountain Sports, Transilvania University of Brasov, Brasov, Romania; ^3^Department of Biostatistics, and Medical Informatics, Faculty of Medicine, Inonu University, Malatya, Türkiye; ^4^Department of Teacher Education, NLA University College, Oslo, Norway

**Keywords:** Mediterranean diet adherence, quality of life, alcohol, tobacco, gender

## Abstract

**Background:**

Adolescence is a key life stage in human development. It is during this stage of development that healthy and physical behaviors are acquired that will last into adulthood. Gender differences in the acquisition of these behaviors have been observed. This research aims to (a) study the levels of Mediterranean diet adherence, quality of life and alcohol and tobacco consumption as regarding the gender of the participants and (b) study the effects of the variable adherence to the Mediterranean diet, alcohol consumption and tobacco consumption on quality of life as a function of the gender of the participants.

**Methods:**

A non-experimental, cross-sectional, exploratory study was carried out in a sample of 1,057 Spanish adolescents (Average Age = 14.19; Standard Deviation = 2.87).

**Results:**

The comparative analysis shows that the male teenagers shows a higher Mediterranean diet adherence compared to the male adolescents (*p* ≤ 0.05) and a higher consumption of alcoholic beverages (*p* ≤ 0.05). On the contrary, adolescent girls show a higher consumption of alcoholic beverages than male participants (*p* ≤ 0.05). The exploratory analysis indicates that for boys, alcohol consumption has a beneficial effect on the quality of life of adolescents (*β* = 0.904; *p* ≤ 0.001).

**Conclusion:**

In this case, participants show differences in the levels of Mediterranean diet adherence, consumption of harmful substances and quality of life according to gender. Likewise, there are different effects between the variables according to gender. Therefore, gender is a key factor to consider during adolescence.

## Background

The current area of study of human development focuses on expanding knowledge of the different phases of the human life cycle (1) and understanding the different changes that occur at each stage (2). Specifically, the adolescent stage takes place between the ages of fourteen and twenty-one and is divided into three phases: Early Adolescence (11–14 years), Middle Adolescence (15–17 years) and Late Adolescence (18–21 years) (3). Variations in the physical and health pattern of adolescents have been observed during the different stages that make up adolescence (3). During adolescence, young people tend to be more sedentary and have a higher intake of high-calorie ready meals (4). Gender differences have also been observed during this stage of human development (5). This has meant that gender is a key factor in understanding physical-healthy behaviors during adolescence (5). Studies have found that there are gender differences in adolescence when it comes to leading an active and healthy lifestyle (6, 7). These differences are often due to changes in food and physical preferences that originate in adolescence (7).

There are numerous variations in the dietary pattern followed during adolescence (8). Throughout the 21st century, unhealthy eating habits have become increasingly prevalent (9). This is mainly due to the easy access adolescents have to this type of high-calorie fast food (9). This results in increased rates of overweight and obese adolescents (10). This concern has led to an increase in the number of adults with metabolic and cardiovascular diseases (10). In terms of nutritional patterns, it has been observed that males are more likely to develop negative behavior toward following a healthy dietary pattern (11). Specifically, within the Mediterranean area, it has been observed that European adolescents show a worse Mediterranean diet adherence (12). A positive follow-up to the Mediterranean diet has reported numerous benefits in health such as reduced prevalence of metabolic syndrome, improved high-density lipoprotein cholesterol among others (13). Psychological improvements have also been observed, such as improvements in executive functions and improvements in the different dimensions of self-concept (13, 14). This dietary pattern is branded by the intake of foods characteristic of the Mediterranean area such as olive oil, vegetables, fruits, oily fish and dairy products (13). In addition, it offers a low intake of processed red meat, saturated fats and low consumption of alcoholic drinks (13).

An intensification in the consumption of alcoholic drinks in the adolescent population has been observed for both males and females (15). The main reason for increased drinking in the adolescent population is related to social recognition (16). A shift in drinking trends has been observed to be taking place (16). This is related to lifestyle, as many adolescents tend to consume alcoholic beverages after physical-recreational activity (17). Physical-recreational activities are becoming a justified cause for drinking alcoholic beverages (18). Regular consumption of alcoholic beverages during adolescence has been shown to lead to decreased academic performance, impaired executive functions and damage to brain cells (18). It has also been observed that young people who drink alcoholic beverages at an early age tend to develop a dependence process toward this substance (19). It has been observed that the consumption of alcoholic beverages can act as a catalyst for the consumption of other harmful substances (19), such as tobacco. It has been found that young people who regularly drink alcoholic beverages show higher tobacco consumption (20). Research has shown that the main reasons why adolescents smoke are pleasure, family influence and social recognition (21). Regular tobacco use has been shown to cause laryngeal cancer, oropharyngeal cancer, lung cancer, myocardial infarction, and stroke (20). This is why regular tobacco and alcohol consumption puts young people’s adult life at risk and worsens their quality of life (21).

Quality of life has been studied under a multidimensional view relating to the social, emotional, mental and physical domains (22). In view of the new patterns of life, it is necessary to study the quality of life of adolescents, as this variable is positively related to the state of health of individuals and to higher academic performance (23). Studies show that the acquisition and promotion of healthy attitudes that are positively related to quality of life are associated with adolescents’ educational attainment (23). This is why education based on the benefits of an active and healthy lifestyle can help to improve the quality of life of adolescents.

Thus, the different implications of this research study in relation to future lines of research focusing on the implementation of health promotion strategies during adolescence stand out. Several descriptive studies have been conducted examining adherence to the Mediterranean diet, quality of life and alcohol and tobacco consumption (9, 11, 17, 20, 22, 23). Despite this, little research has been found that focuses on studying the effects of these variables through multigroup equation modeling. That is why this study aims:

- To study the levels of adherence to the Mediterranean diet, quality of life and alcohol and tobacco consumption according to the gender of the participants.
- To study the effects of the variable adherence to the Mediterranean diet, alcohol consumption and tobacco consumption on quality of life according to the sex of the participants.

## Methods

### Design and participants

In line with the proposed objectives, the design of this research is non-experimental (*ex post facto*), comparative, exploratory and cross-sectional (only a single measurement was taken). It should be noted that the socio-economic level of the families is moderate and that half of the parents have a university education.

Moving on to the research participants, the sample of this study consists of 1,057 (Average Age = 14.19; Standard Deviation = 2.87) secondary school students. The population consists of 547 boys (51.75%) and 510 girls (48.25%). Following Byrnes et al. (24) and Melguizo-Ibáñez et al. (25), adolescence is made up of three stages: Early adolescence (11–14 years), middle adolescence (15–17 years) and late adolescence (18–21 years). According to this classification, 918 belong to early adolescence, 139 belong to middle adolescence and none to late adolescence. Continuing with the study of the sampling error, for a confidence level of 95.0%, a level of less than 5.0% was obtained.

### Instruments

The following instruments were used to collect the data:

*Own sociodemographic questionnaire*: It was employed to collect sociodemographic data like age and gender (male/female).

*Alcohol Use Disorders Identification Test (AUDIT)* (26): It has been used the Spanish version (27). It is made up of 10 items. The first eight are assessed using a 5-choice Likert scale (0 = Never; 4 = Daily). The last items are assessed using a 3-option Likert scale. Regarding the reliability analysis, a value of α = 0.749 was obtained.

*Fagerström Test for Nicotine Dependence (FTND)* (28): Due to the characteristics of the sample, the Spanish version was used (29). This questionnaire provides information on the number of cigarettes smoked by the subject, the urge to smoke and the degree of dependence on nicotine. It consists of 6 items. Cronbach’s alpha obtained a value of α = 0.916.

*Mediterranean Diet Adherence Test (KIDMED)* (30): This questionnaire is used to determine the degree of adherence to the Mediterranean diet. This questionnaire is made up of 16 questions. These questions are answered positively or negatively. The instrument presents four questions written in a negative way, so that if they are answered positively, they are valued with −1 point. The twelve questions, if answered positively, are rated with +1 point. Cronbach’s alpha evidenced a value of *α* = 0.702.

*Quality of Life*: The short version of the SF-36 questionnaire (31) was used to measure this variable. Specifically, we used the version adapted to Spanish (32). This instrument examines two distinct areas. The first dimension assesses the dimensions of bodily pain, general health, role and physical function. The second assesses the areas of vitality, social function and emotional role. Cronbach’s alpha obtained a value of *α* = 0.916.

### Procedure

Initially, a reading of the different research on this subject was carried out. Based on these readings, the most reliable instruments were selected and this study was theoretically contextualized. In order to carry out the fieldwork, three secondary schools were randomly selected. The schools were then contacted and informed of the aims of the study. Once informed, the schools sent an informative letter to the different teenagers’ legal guardians. Once the legal guardians were informed of the research objectives, they were asked to sign the informed consent form, authorizing their children to participate. In this informed consent, the legal guardians were notified that data would be processed scientifically and in an anonymized form.

During data collection, the researchers were always present to answer any questions. All data were collected during physical education classes. This was done so as not to interrupt the teaching of other subjects.

With regard to the ethical aspects related to research ethics, this study followed the criteria redacted in the Declaration of Helsinki. Likewise, throughout the research process, the study was supervised by an ethics committee of the University of Granada (2966/CEIH/2022).

### Data analysis

Initially, the normality of the data was checked with the Kolmogorov–Smirnov test with Lillieforts correction. Homoscedasticity was measured using Lev’s test. The variables followed a non-normal distribution. The analyses were carried out using non-parametric tests. For the comparative analysis, the Mann–Whitney U test was used. The significance level was set at *p* ≤ 0.05. The statistical program IBM SPSS 25.0 for Windows was used to perform all the above.

**Figure 1:**
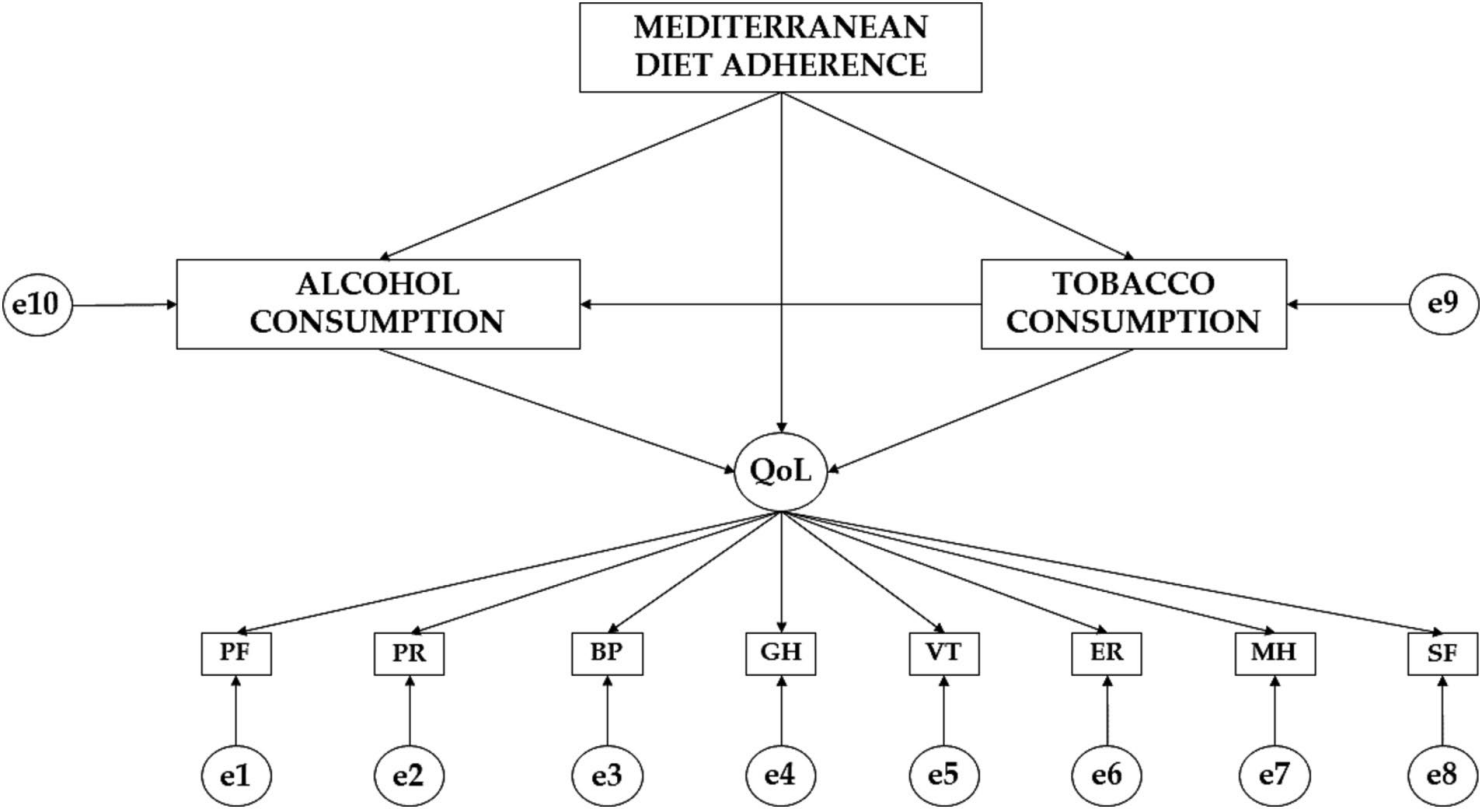
Theoretical model proposed. PF, physical function; PR, physical role; BP, bodily pain; GH, general health; VT, vitality; SF, social function; ER, emotional role; MH, mental health.

Continuing with the exploratory analysis, the theoretical model (Figure 1) consists of twelve variables. Ten variables play an endogenous role and two variables act as exogenous variables. For the variables acting as endogenous, causal explanations have been made. These have been done by taking into account the observed associations between the degree of reliability of the measurement process and the indicators. This allows measurement error to be included in the different models. Furthermore, measurement error can be controlled and interpreted as multivariate regression coefficients. The direction of the arrows refers to the direction in which the effect occurs. In this case the significance level was set at *p* ≤ 0.05 and *p* ≤ 0.001. IBM SPSS Amos 26.0 (IBM Corp., Armonk, NY, USA) was used to develop the theoretical model.

To assess the fit of the theoretical model, the established criteria have been followed (33, 34). Initially, the fit of the Chi-Square test should be taken into account. When non-significant values are obtained, a good fit is evident. The following indices should also be considered: Comparative Fit Index (CFI), Goodness of Fit Index (GFI) and Incremental Fixity Index (IFI). The values must be higher than 0.90 to obtain a good fit. Also, the Root Mean Square Approximation (RMSEA) value must be taken into account. For this index values should be less than 0.100. Following the criteria established by Tenembaum and Eklund (35), sample size and susceptibility should be considered. Therefore, the Tucker Lewis Index has been included, which should be equal to or greater than 0.900.

The proposed model for the male adolescents obtained a non-significant value (Chi-Square = 78.760; Degrees of Freedom = 19; Probability Level = 0.002). The fit indices obtained an excellent fit (CFI = 0.965; IFI = 0.974; TLI = 0.952; RMSEA = 0.068). The proposed model for the female population obtained a non-significant value (Chi-Square = 32.726; Degrees of Freedom = 20; Probability Level = 0.008). The fit indices obtained an excellent fit (CFI = 0.976; IFI = 0.964; TLI = 0.939; RMSEA = 0.081).

## Results

Table 1 shows the results obtained from the comparative analysis. It is observed that males shows higher levels in physical functionality (PF) (2.9653 ± 0.09468 vs. 2.9394 ± 0.14593; *p* ≤ 0.05), physical role (PR) (1.9073 ± 0.25821 vs. 1.9016 ± 0.26382), social function (SF) (3.4672 ± 0.43987 vs. 3.4173 ± 0.53364; *p* ≤ 0.05) emotional role (ER) (1.8728 ± 0.28883 vs. 1.8163 ± 0.36541; *p* ≤ 0.05), mental health (MH) (3.9092 ± 0.37755 vs. 3.8693 ± 0.38740), Mediterranean diet adherence (0.8008 ± 0.08255 vs. 0.7930 ± 0.07939; *p* ≤ 0.05) and tobacco consumption (1.5450 ± 0.74462 vs. 1.402929 ± 0.71684; *p* ≤ 0.05). On the contrary, female teenagers show higher scores in bodily pain (2.0551 ± 1.02591 vs. 1.8957 ± 0.98256), general health (GH) (3.0110 ± 0.39626 vs. 2.9703 ± 0.35236; *p* ≤ 0.05), vitality (VT) (3.4350 ± 0.48306 vs. 3.4258 ± 0.51263) and alcohol consumption (4.0661 ± 0.57752 vs. 4.0235 ± 0.59279; *p* ≤ 0.05).

**Table 1:** Comparative analysis of the sample.

		*N*	*M* ± S.D	*P*
Physical function	Female	127	2.9394 ± 0.14593	0.007
	Male	930	2.9653 ± 0.09468	
Physical role	Female	127	1.9016 ± 0.26382	0.817
	Male	930	1.9073 ± 0.25821	
Bodily pain	Female	127	2.0551 ± 1.02591	0.880
	Male	930	1.8957 ± 0.98256	
General health	Female	127	3.0110 ± 0.39626	0.029
	Male	930	2.9703 ± 0.35236	
Vitality	Female	127	3.4350 ± 0.48306	0.089
	Male	930	3.4258 ± 0.51263	
Social function	Female	127	3.4173 ± 0.53364	0.025
	Male	930	3.4672 ± 0.43987	
Emotional role	Female	127	1.8163 ± 0.36541	0.039
	Male	930	1.8728 ± 0.28883	
Mental health	Female	127	3.8693 ± 0.38740	0.087
	Male	930	3.9092 ± 0.37755	
Mediterranean diet adherence	Female	127	0.7930 ± 0.07939	0.039
	Male	930	0.8008 ± 0.08255	
Alcohol consumption	Female	127	4.0661 ± 0.57752	0.043
	Male	930	4.0235 ± 0.59279	
Tobacco consumption	Female	127	1.4029 ± 0.71684	0.039
	Male	930	1.5450 ± 0.74462	

**Figure 2:**
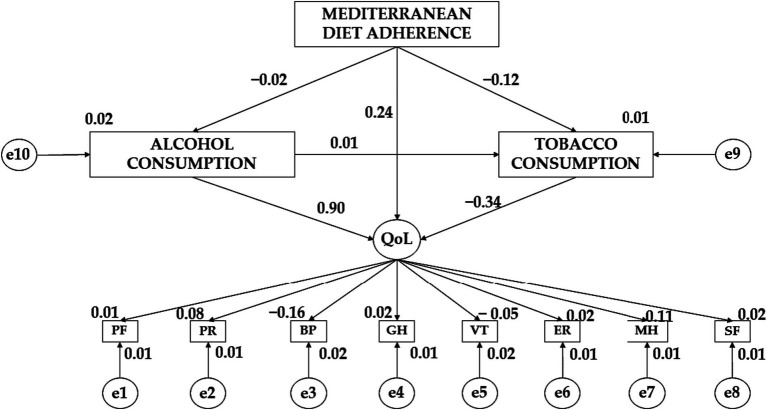
Theoretical model together with standardized regression weights for the male gender.

Table 2 together with Figure 2 show the standardized regression weights for boys. Regarding the relationship between adherence to the Mediterranean diet (MDA) and alcohol consumption (AC), a negative effect is observed (*β* = −0.025). There was also a negative effect of Mediterranean diet adherence (MDA) on tobacco consumption (TC) (*β* = −0.120; *p* ≤ 0.05). A positive effect of alcohol consumption (AC) on tobacco consumption (TC) was observed (*β* = 0.004). Continuing with the effect of alcohol consumption (AC) on quality of life (QoL), a positive impact is obtained (*β* = −0.904; *p* ≤ 0.001). Likewise, a positive effect of Mediterranean diet adherence (MDA) on quality of life (QoL) is obtained (*β* = 0.245). In contrast, a negative impact of tobacco consumption (TC) on quality of life (QoL) was obtained (*β* = −0.344; *p* ≤ 0.05).

**Table 2:** Male standardized regression weights.

Effect direction	Regression weights				Standardized regression weights
	Estimations	Estimation error	Critical Ratio	*p*	Estimations
AC ← MDA	−0.176	0.236	−0.748	0.454	−0.025
TC ← MDA	−1.084	0.294	−3.689	**	−0.120
TC ← AC	0.005	0.041	0.119	0.905	0.004
QoL ← AC	0.061	0.019	3.191	***	0.904
QoL ← TC	−0.019	0.009	−2.029	**	−0.344
QoL ← MDA	0.119	0.076	1.569	0.117	0.245
MH ← QoL	1.000				0.107
ER ← QoL	0.598	0.298	2.007	**	0.083
SF ← QoL	0.238	0.366	0.651	0.515	0.022
VT ← QoL	−0.642	0.462	−1.390	0.164	−0.050
GH ← QoL	0.145	0.291	0.499	0.618	0.017
BP ← QoL	−3.895	1.432	−2.721	***	−0.159
PR ← QoL	0.536	0.267	2.012	**	0.084
PF ← QoL	0.011	0.077	0.145	0.885	0.005

**p < 0.05.***p < 0.001.

Considering the variables that make up the Quality-of-Life (QoL) variable, a negative effect of this variable on vitality (VT) (*β* = − 0.050) and bodily pain (BP) (*β* = −0.159; *p* ≤ 0.001) is obtained. On the contrary, a positive effect of Quality-of-Life (QoL) on mental health (MH) (*β* = 0.107), emotional role (ER) (*β* = 0.083; p ≤ 0.05), social function (SF) (*β* = 0.022), general health (GH) (*β* = 0.017), physical role (PR) (*β* = 0.084; p ≤ 0.05) and physical function (PF) (*β* = 0.005) is observed.

**Figure 3:**
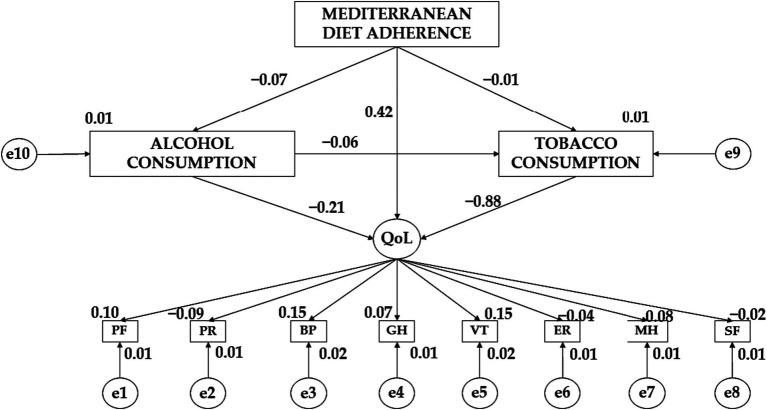
Theoretical model together with standardized regression weights for the female gender.

Table 3 together with Figure 3 show the standardized regression weights for girls. In this case, Mediterranean diet adherence (MDA) has a negative effect on alcohol consumption (AC) (*β* = −0.072) and tobacco consumption (TC) (*β* = −0.011) consumption. Similarly, alcohol consumption (AC) has a negative effect on tobacco consumption (TC) (*β* = −0.055) and quality of life (QoL) (*β* = −0.208). In contrast, Mediterranean diet adherence (MDA) has a positive effect on quality of life (QoL) (*β* = − 0.425). Continuing with the variables that make up quality of life (QoL), it is observed a negative effect of this variable on emotional role (ER) (*β* = − 0.036), social function (SF) (*β* = − 0.018) and physical role (PR) (*β* = − 0.087). In contrast, quality of life (QoL) exerts a positive effect on vitality (VT) (*β* = 0.155), general health (GH) (*β* = − 0.072), bodily pain (BP) (*β* = 0.145) and personal role (PR) (*β* = 0.101).

**Table 3:** Female standardized regression weights.

Effect direction	Regression weights				Standardized regression weights
	Estimations	Estimation error	Critical Ratio	Estimations	Estimation error
AC ← MDA	−0.527	0.646	−0.815	0.415	−0.072
TC ← MDA	−0.103	0.805	−0.127	0.899	−0.011
TC ← AC	−0.069	0.111	−0.622	0.534	−0.055
QoL ← AC	−0.011	0.021	−0.533	0.594	−0.208
QoL ← AC	−0.038	0.043	−0.885	0.376	−0.882
QoL ← MDA	0.165	0.216	0.763	0.445	0.425
MH ← QoL	1.000				0.080
ER ←QoL	−0.429	1.159	−0.370	0.711	−0.036
SF ← QoL	−0.305	1.579	−0.193	0.847	−0.018
VT ← QoL	2.429	3.042	0.798	0.425	0.155
GH ← QoL	0.925	1.540	0.601	0.548	0.072
BP ← QoL	4.842	6.150	0.787	0.431	0.145
PR ← QoL	−0.746	1.127	−0.662	0.508	−0.087
PF ← QoL	0.478	0.678	0.704	0.482	0.101

## Discussion

The aims of this research are to study the levels of Mediterranean diet adherence, quality of life and alcohol and tobacco consumption according to the gender of the participants and to study the effects of the variable adherence to the Mediterranean diet, alcohol consumption and tobacco consumption on quality of life according to the gender of the participants. Once the relevant data analysis has been carried out to meet these objectives, the discussion aims to compare the results obtained with those of other similar studies.

With regard to the comparative analysis, it is observed that boys show greater adherence to the Mediterranean diet. Despite these results, research indicates that the male adolescents show a greater degree of complexity when it comes to following a healthy dietary pattern (36). Studies have found that there are no statistically significant differences between adherence to the Mediterranean diet and gender (37, 38). It has been observed that women tend to pay more attention to the type of food they eat and how it is cooked (38). A decline in adherence to the Mediterranean diet has also been observed in adolescents in Greece, Italy and Spain (39). This is due to new food trends where, through new food apps, speed is prioritized over food quality and cooking (39). There are also factors that condition the degree of adherence to the Mediterranean diet, such as the socioeconomic level of families and the parents’ level of education (40).

Continuing with the consumption of alcohol and tobacco, it is observed that female teenagers show a higher consumption of alcohol and the boys show a higher consumption of tobacco. In view of these findings, it has been pointed out that in Europe a higher intake of harmful substances is taking place at earlier ages of human development (16). Research similar to the present study found no statistically significant differences according to gender (17). Despite these findings, it has been found that males are more likely to exhibit positive alcohol and tobacco behaviors throughout adolescence (17). Alcohol and tobacco use data have been positively related to social recognition and peer image (41). It has been shown that males attach greater importance to social recognition during adolescence, thus favoring unhealthy behaviors (17).

Continuing with the quality of life, it is observed that the male population shows higher scores in physical function, physical role, social function, emotional role, and mental health. In contrast, males show higher scores in vitality and general health. It has been observed that during adolescence the female gender shows a lower weekly physical activity time (17, 38). Studies indicate that males are more active than females during adolescence (17, 38). It has been observed that regular physical exercise has benefits for the physical function of the body, emotional control, social and mental health (13). In the physical area, physical activity helps to reduce body mass index, burn fat and improve aerobic capacity (13). In terms of emotional control and improved mental health, physical activity promotes the release of neurotransmitters such as serotonin and dopamine (42). It has also been observed that an active lifestyle helps to prevent the onset of body aches and pains resulting from physical inactivity (4, 13). Physical activity helps to improve general health (4). However, there are elements that have a negative impact on vitality and general health, such as alcohol, tobacco and cannabis consumption (17). In this case, it has been observed that the girls are reluctant to ingest these substances (17).

The exploratory analysis shows a negative effect of adherence to the Mediterranean diet on alcohol and tobacco consumption for male and female population. In this case, the Mediterranean diet has been found to be a dietary pattern that provides health benefits (13). This dietary pattern is characterized by a low intake of alcoholic beverages (13). Tobacco use has been reported to have negative effects on the health of individuals, irrespective of gender (16, 18). It has been observed that the boys are more prone to the intake of these substances as they help in acquiring greater social recognition from their peers (17).

Continuing with the effect of alcohol consumption on tobacco consumption and quality of life, the female teenagers show a negative effect. In contrast, males show an effect of alcohol consumption on tobacco consumption and quality of life. Studies indicate that alcohol consumption at an early age favors the use of tobacco and other psychoactive substances (17, 19, 20). The study by Jacobs et al. (43) states that girls show a greater rejection of alcohol and tobacco use. Regular consumption of alcoholic beverages is shown to worsen the quality of life of young people (43). However, there is an increasing intake of alcoholic beverages at younger and younger ages (43, 44). This may be due to a lack of awareness of the effects of alcohol on quality of life (44). It has also been reported that alcohol is used as a socializing element, thus encouraging socializing among peers (17, 44).

There is also a positive effect of adherence to the Mediterranean diet on quality of life for both men and women. Positive adherence to this dietary pattern has been observed to have benefits in different spheres of the human being (13). In this case, benefits have been observed at the organ level, such as reduced blood pressure and increased life expectancy (13). At the mental level, improvements in executive functions and improvements in self-concept have been found (45).

### Strengths and limitations

In terms of the strengths of this study, it is worth noting its applicability. This research offers totally reliable data through various analyses where the current state of the variables is described and where the effect of these variables is analyzed according to gender. In this case, this study highlights the existing differences between the male and female sexes when it comes to leading a healthy lifestyle. When designing intervention programs, the motivations between men and women should be taken into account.

Finally, this research is not without limitations. The first of these is related to the design of the study. Being a cross-sectional study, data were only collected once. Although the instruments have been validated and adapted by the scientific community, they have an intrinsic measurement error. For future research it would be interesting to add new study variables. It would be advisable to add variables related to the socio-economic level of the families. It would also be advisable to add variables related to academic performance.

## Conclusion

The conclusions obtained from this research are shown below.

In terms of objective one, it is concluded that boys show a greater adherence to the Mediterranean diet and a higher consumption of tobacco. On the other hand, the female adolescents show a higher consumption of alcoholic beverages. In terms of quality of life, the male teenagers show higher levels in the variables that make up this variable.

Continuing with the second objective, it is concluded that there are considerable differences in the relationship between the effect of the Mediterranean diet and the consumption of alcohol and tobacco on quality of life according to the gender of the participants.

As a general conclusion, this study shows that during adolescence, gender is a key factor when it comes to leading a healthy lifestyle.

## Data availability statement

The raw data supporting the conclusions of this article will be made available by the authors, without undue reservation.

## Ethics statement

The study was conducted according to the guidelines of the Declaration of Helsinki and approved by the Institutional Review Board and approved by the Ethics Committee of University of Granada under Statement Number 2966/CEIH/2022. Written informed consent was obtained from all individual participants included in the study.

## Author contributions

EM-I: Conceptualization, Data curation, Formal analysis, Investigation, Methodology, Resources, Supervision, Validation, Visualization, Writing – original draft, Writing – review & editing. FZ-O: Conceptualization, Data curation, Formal analysis, Investigation, Methodology, Writing – original draft, Writing – review & editing. JU-J: Data curation, Methodology, Validation, Writing – original draft, Writing – review & editing. GB: Formal analysis, Methodology, Supervision, Validation, Visualization, Writing – original draft, Writing – review & editing. FY: Supervision, Validation, Writing – original draft, Writing – review & editing. GG-V: Investigation, Methodology, Validation, Visualization, Writing – original draft, Writing – review & editing. LPA: Supervision, Validation, Writing – original draft, Writing – review & editing. PP-M: Methodology, Project administration, Supervision, Validation, Writing – original draft, Writing – review & editing.
